# Multihydrophone Fusion Network for Modulation Recognition

**DOI:** 10.3390/s22093214

**Published:** 2022-04-22

**Authors:** Haiwang Wang, Bin Wang, Lulu Wu, Qiang Tang

**Affiliations:** School of Information Systems Engineering, PLA Strategic Support Force Information Engineering University, Zhengzhou 450001, China; oceanw_ang@163.com (H.W.); pstgrdt2021@163.com (L.W.); thouash@163.com (Q.T.)

**Keywords:** multihydrophone, modulation recognition, underwater acoustic communication signal, fusion network

## Abstract

Deep learning (DL)-based modulation recognition methods of underwater acoustic communication signals are mostly applied to a single hydrophone reception scenario. In this paper, we propose a novel end-to-end multihydrophone fusion network (MHFNet) for multisensory reception scenarios. MHFNet consists of a feature extraction module and a fusion module. The feature extraction module extracts the features of the signals received by the multiple hydrophones. Then, through the neural network, the fusion module fuses and classifies the features of the multiple signals. MHFNet takes full advantage of neural networks and multihydrophone reception to effectively fuse signal features for realizing improved modulation recognition performance. Experimental results on simulation and practical data show that MHFNet is superior to other fusion methods. The classification accuracy is improved by about 16%.

## 1. Introduction

Modulation recognition of underwater acoustic communication signals, which is the key link of underwater information acquisition, refers to the process of accurately classifying the signal modulation mode under the condition of a known modulation typeset. With the upgrade of hardware, modulation recognition based on sensor networks has become a common method of communication. In the field of radio modulation recognition, researchers agree that the modulation recognition performance of multiple sensors is better than that of a single sensor [1,2,3,4]. Modulation recognition methods based on sensor networks can be divided into signal fusion [1], feature fusion [2], and decision fusion [3,4]. The signal fusion method is more sensitive to the delay difference and the synchronization error of sensor nodes. Both feature fusion and decision fusion methods use sub-node preclassification and main node fusion classification. These methods are mainly based on likelihood ratios [3] and higher-order statistical features of signals [1]. Among them, the recognition performance of the likelihood ratio method reaches the theoretical upper limit under the condition of infinite available sequences. However, the method faces problems, such as the complexity of the Bayesian threshold calculation and the need for prior information. Classification methods based on higher-order statistics have problems, such as poor feature generalization and long signal sequences. In addition, the master node fusion method can be categorized into signal-to-noise ratio-based weight assignment [3] and voting [4] methods. These methods, however, have problems, such as a complex algorithm process, inaccurate estimation of the signal-to-noise ratio, and an inflexible fusion mechanism.

**Figure 1:**
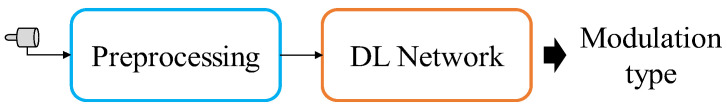
Basic flow of a single hydrophone modulation recognition method based on DL.

In recent years, deep learning (DL) has made major breakthroughs in the modulation recognition of underwater acoustic communication signals by virtue of its powerful feature extraction and computing capabilities. The current research based on DL can be divided into two categories—the modulation recognition method applied to a single hydrophone scenario and that applied to a multihydrophone scenario. Various network structures have been designed for the single hydrophone modulation recognition problem to realize the effective recognition of underwater acoustic communication signals. Figure 1 shows its basic flow.

First, the received signal is preprocessed, and then different network structures are used to complete the feature extraction and classification of the signal. Common preprocessing includes noise reduction processing [5,6], filtering, and selection of shallow features. Shallow feature selection involves the conversion of signal sequences into a form suitable for network recognition, such as signal power spectrograms [6], in-phase/quadrature (I/Q) sequences [7], time–frequency diagrams [8], and multimodal features [9]. Different DL networks are selected for different shallow features. Common DL networks include the long short-term memory network [10], convolutional neural network (CNN) [11], capsule network [3], generative adversarial network [8], autoencoder network [12], and residual network [13]. With the combination of different DL networks and shallow features, DL-based methods have made considerable progress in the field of modulation recognition.

Unlike the single hydrophone scenario, multiple hydrophones can use different locations to obtain signals with different reception qualities. Then, a fusion strategy is adopted to improve the reliability of signal recognition. Due to the lack of literature on multihydrophone modulation recognition, we refer to the signal recognition method based on multi-antenna reception. Wang et al. [14] proposed a DL modulation recognition method applied to multiple-input multiple-output (MIMO) systems. This method uses CNNs to extract the signal characteristics of different receiving sources, and then it uses direct voting (DV) and direct averaging (DA), weighty voting (WV), and weighty averaging (WA) fusion classification of the recognition results of different receiving sources. Among them, WV and WA methods require the prior information of the receiving antenna; this requirement cannot be satisfied in the problem of noncooperative modulation recognition. Similarly, An et al. [15] proposed a cooperative modulation recognition method based on the one-dimensional CNN for MIMO orthogonal space–time block code signal. This method trains different DL networks according to the receiving antenna, and the network structure is complex. In addition, the voting fusion and confidence decision fusion methods have low intelligence, and the weight distribution of different decision results is inflexible, ignoring the quality of signals at different receiving locations. Overall, for radio MIMO systems, researchers have used the traditional information fusion decision mechanism to improve performance. However, such a mechanism ignores the difference in the location of the receiving source, and the weight distribution is not flexible. Thus, the performance of the modulation recognition method is worth further study. In addition, compared with radio communication, underwater acoustic communication often faces more serious problems, such as fading, Doppler shift, multipath effects, and fluctuation effects. Therefore, the multi-antenna fusion recognition method in the radio field cannot be easily applied to underwater acoustic communication.

In this paper, by considering the combination of the actual underwater acoustic communication scenarios and the limitations of the fusion decision-making mechanism in the radio field, we introduce a multihydrophone fusion network (MHFNet) for reception modulation recognition. The specific contributions of the study are as follows.

1. This study proposes a new network framework, including a feature extraction module and a fusion module, which successfully realizes effective identification in the multihydrophone reception scenarios of 2FSK, 4FSK, 8FSK, BPSK, LFM, and OFDM and other commonly used underwater acoustic communication signals.
2. To extract the characteristics of the signals received by multiple hydrophones, this study uses a CNN to extract the characteristics of the signal time-domain sequence.
3. To fully utilize different locations to obtain signals of different reception qualities, this study uses a neural network to construct a fusion module that can automatically evaluate the signal quality and assign the optimal weights to different hydrophones.
4. Experimental results show that the proposed method is better than DV and DA in multiple hydrophone scenarios, showing an improvement of approximately 16% when the symbol signal-to-noise ratio is 10 dB.

## 2. System Model

**Figure 2:**
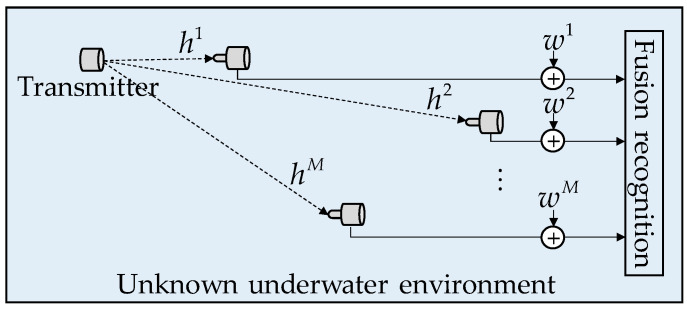
Modulation recognition method based on deep learning in multiple reception scenarios.

This study considers a multihydrophone receiver system, shown in Figure 2. First, to receive signals from unknown underwater acoustic communication sound sources, multiple hydrophones are deployed at different locations in the target sea area. Then, MHFNet is adopted for feature fusion and recognition of the signals received from different locations by multiple hydrophones.

In the signal receiving process, owing to the lack of a priori information of the underwater acoustic communication source, the location of hydrophones is random, and the relative distance and water environment are unknown. Therefore, the different underwater acoustic transmission channels are assumed to be independent of each other. The signal models received by different hydrophones are modeled as

(1) R=H∗s(n)+W,

where $R={\left[r^{1}(n),r^{2}(n),\cdots ,r^{M}(n)\right]}^{T}$ represents the signal received by M hydrophones, $H={\left[h^{1}(n),h^{2}(n),\cdots ,h^{M}(n)\right]}^{T}$ represents the impulse response of M independent underwater acoustic channels, and s(n) is the transmitted signal. The modulation types of the signal include: 2FSK, 4FSK, 8FSK, BPSK, LFM, and OFDM. $W={\left[w^{1}(n),w^{2}(n),\cdots ,w^{M}(n)\right]}^{T}$ represents marine environmental noise, which is modeled as Gaussian distributed noise in this paper.

## 3. Proposed Recognition Approach

**Figure 3:**
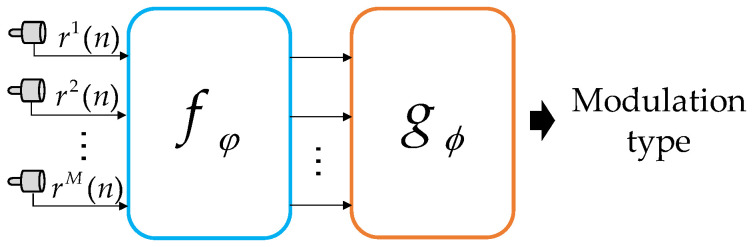
Structure of MHFNet.

The structure of MHFNet is shown in Figure 3, including the feature extraction module $f_{\varphi }$ and fusion module $g_{\phi }$. First, the signals from multiple hydrophones are fed to the feature extraction module to obtain high-dimensional feature maps. Then, a fusion module is designed to fuse these maps. Finally, the recognition of signal types is completed on the basis of the difference in fusion features.

### 3.1. Feature Extraction Module

In this paper, the sample matrix composed of the received signals of multiple hydrophones is fed to the feature extraction module. The signal sample matrix R can be expressed as

(2) $$R=\left[\begin{array}{l} r^{1}(n)\\ r^{2}(n)\\ \cdots \\ r^{M}(n) \end{array}\right]=\left[\begin{array}{l} r^{1}(0),\;\ldots ,\;r^{1}(L-1)\\ r^{2}(0),\;\ldots ,\;r^{2}(L-1)\\ \cdots \\ r^{M}(0),\;\ldots ,\;r^{M}(L-1) \end{array}\right],$$

where M represents the number of hydrophones and L represents the number of sampling points of the signal.

**Figure 4:**
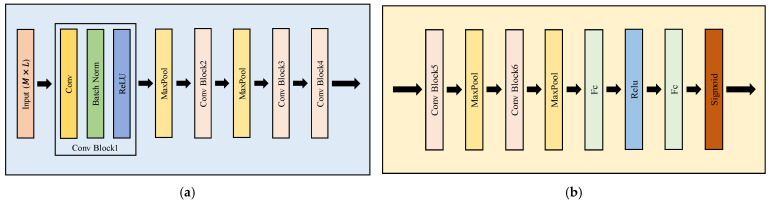
Network structure of MHFNet. (**a**) Network structure of the feature extraction module; (**b**) network structure of the fusion module.

The feature extraction module is composed of four convolution (conv) blocks and two pooling layers. The specific structure is shown in Figure 4a. In each conv block, first, two-dimensional convolution is used to extract the features of the input sample matrix R. The size of each convolution filter is 1 × 31, and the number of convolution kernels is 64. Then, batch normalization is adopted to enhance stability and reduce the interaction between the following layers. A rectified linear unit (ReLU) is utilized as the activation function. In addition, a 1 × 2 max-pooling layer is used after the first two conv blocks for further signal extraction. Overall, the high-dimensional feature maps $f_{\varphi }\left(R\right)$ of the multiplex signal are obtained by convolution, pooling, and other operations.

### 3.2. Fusion Module

In the problem of modulation recognition of noncooperative underwater acoustic communication signals, because of the lack of prior knowledge, the quality of signals from different hydrophones is different. Because DV and DA ignore the analysis of the signal quality of different hydrophones, there is significant room for improvement in performance. The key concept of the fusion module is to use the self-learning ability and computing power of the neural network to automatically evaluate the signal quality from different hydrophones and assign the best weight to each hydrophone. Equivalently, a hydrophone with a high signal-to-noise ratio is assigned a large weight, while that with a low ratio is assigned a small weight. It makes the fused signal features more discriminative. The network structure of the fusion module is shown in Figure 4b. First, the feature maps’ output from the feature extraction module are fed to the fusion module. Then, two conv blocks, two max-pooling layers, two fully connected layers (Fc), and a sigmoid activation function are used to fuse the feature maps of the received signals. The final output is a 1 × 6 matrix $P={\left\{p_{j}\right\}}_{j=1}^{C}$, where C indicates the number of underwater acoustic communication signal categories, which is 6 in this paper. The matrix element is denoted as $p_{j}=g_{\phi }[f_{\varphi }(R)]$.

### 3.3. Loss Function

The loss function is designed as follows:

(3) $$\varphi ,\;\phi \leftarrow \arg\min\sum_{i=1}^{T}\sum_{j=1}^{C}{\left(p_{i,j}-1(y_{i}=\;=y_{j})\right)}^{2},$$

where φ and ϕ are the network parameters of the feature extraction module and the fusion module, respectively; T is the number of signal samples received by a single hydrophone; $p_{i,j}$ represents the probability that the i-th test signal belongs to the j-th type of signal, and its range is [0, 1]; $y_{i}$ is the true label of the signal; and $y_{j}$ is the predicted label, which is the recognition result of MHFNet. We achieve the optimization of parameters φ and ϕ of MHFNet by using loss functions. The optimal modulation recognition network is finally obtained.

## 4. Review of the Other Fusion Method

**Figure 5:**
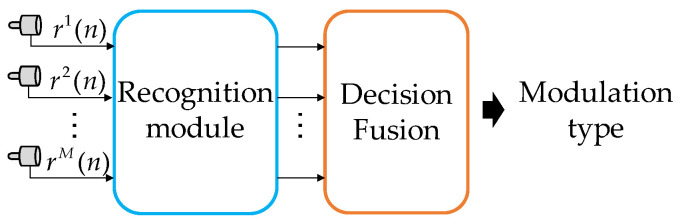
Structure of the decision fusion method.

Information fusion methods include signal fusion, feature fusion and decision fusion. In this study, the feature fusion method is used to fuse multichannel received signals and then identify and classify them. To highlight the advantages of the algorithm, we assess the performance of each of the other fusion methods. The first method is signal fusion. In actual underwater acoustic communication, owing to the effect of multichannel underwater acoustics, the signals received by multiple hydrophones have a large time delay and severe synchronization errors. The signal fusion method is sensitive to the delay and synchronization errors of the signals received by multiple hydrophones; hence, it is not a feasible method. Next, we explore decision fusion. The decision fusion method includes voting and confidence decision methods. References [14,15] proposed a combination of DL networks and decision fusion; the flow of this method is shown in Figure 5.

The signals received by different hydrophones are first subjected to preprocessing, then identified in the recognition module ${\tilde{f}}_{\tilde{\varphi }}$. The output recognition result is a M × 6 matrix $\tilde{P}=\{{\tilde{p}}_{j}^{m}\}$, where j∈[1,C] represents the number of underwater acoustic communication signal categories, m∈[1,M] represents the m-th hydrophone. The matrix element is denoted as ${\tilde{p}}_{j}^{m}={\tilde{f}}_{\tilde{\varphi }}(R)$. The recognition results are input to the decision fusion module, wherein various decision fusion methods are used for fusion recognition to finally obtain the modulation type of the signal.

To compare the fusion performance of different methods and ensure fairness of the experiment, this study adopts a network structure similar to the MHFNet. The recognition module of this study adds two Fc to serve as classifiers based on the feature extraction module of MHFNet. The decision fusion method in this study includes voting fusion and confidence decision fusion, which are equivalent to DV and DA in [14], respectively. In addition, we design the loss function of the decision fusion recognition method based on Section 3.3, and its expression is as follows:

(4) $$\tilde{\varphi }\leftarrow \arg\min\sum_{i=1}^{T}\sum_{m=1}^{M}\sum_{j=1}^{C}{\left({\tilde{p}}_{i,j}^{m}-1(y_{i}=\;=y_{j})\right)}^{2},$$

where $\tilde{\varphi }$ represents the network parameters of the recognition module, ${\tilde{p}}_{i,j}^{m}$ represents the probability that the i-th test signal received by the m-th hydrophone belongs to the j-th type of signal, and its range is [0, 1].

## 5. Performance Analysis

### 5.1. Generation of Underwater Acoustic Communication Signals

In this section, we generate data for network training and testing based on Equation (1). The sampling rate of the signal is 48 kHz, the signal length L is 8192 sampling points, for approximately 171 ms, and the OFDM subcarrier is BPSK or QPSK. Other signal parameters are shown in Table 1.

In the single-input multiple-output (SIMO) system, depending on the receiving location, environment, and distance of different hydrophones, the transmitted signal reaches the receiving end passing through different channels. For a close representation of the actual application scenario, we choose the typical underwater acoustic channel $h_{A}$–$h_{F}$ in [16] as the receiving channel of different hydrophones. In the training process, channels are randomly selected to generate signal samples according to the number of hydrophones. To measure the quality of the received signal, we use the symbol signal-to-noise-ratio, which is defined as the ratio of the energy per symbol to the noise power spectral density, denoted as $E_{s}{/N}_{0}$. We generate 100 samples per $E_{s}{/N}_{0}$, per hydrophone, and per class of signal for training. $E_{s}{/N}_{0}$ is in the range of [0–24] dB, with an interval of 2 dB.

In the testing process, we select channels $h_{A}$, $h_{C}$, and $h_{D}$ with a sound source depth of 200 m and different receiving depths and distances given in [16] as the test channels, and we generate 200 samples per class of signal per hydrophone for the testing. $E_{s}{/N}_{0}$ is in the range of [0–20] dB with an interval of 2 dB.

### 5.2. Experiment Setting

The experiment in this paper is based on the PyTorch DL platform for training and testing in an environment with the following specifications: the CPU is Intel(R) Xeon(R) Silver 4110, the GPU is RTX2080Ti, and the memory is 128 GB. The training process uses the Adam optimizer, and the hyperparameters of MHFNet are set as follows: the learning rate is 0.0005, the batch size is 60 × M, and the training epochs are 4000.

### 5.3. Result Analysis

**Figure 6:**
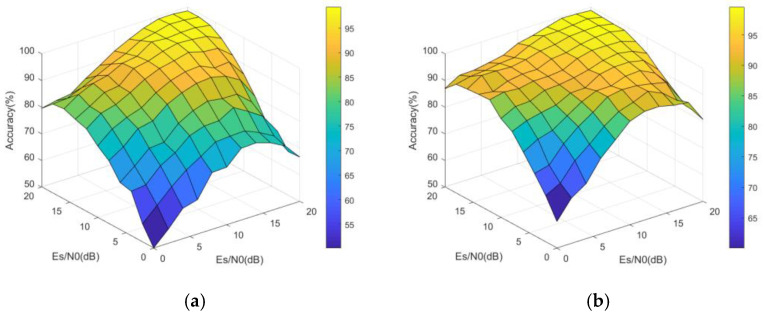
Overall recognition performance. (**a**) $E_{s}{/N}_{0}$ = 0 dB; (**b**) $E_{s}{/N}_{0}$ = 10 dB.

First, the overall recognition performance of MHFNet is analyzed. When M=3, the performance of MHFNet with different hydrophones under different symbol signal-to-noise ratio conditions is tested. Figure 6 shows the visual demonstration of the recognition accuracy of MHFNet, assuming the $E_{s}{/N}_{0}$ value of a hydrophone to be 0 dB and 10 dB while varying the $E_{s}{/N}_{0}$ values of the remaining two hydrophones from 0 to 20 dB, with an interval of 2 dB. The figure indicates that the greater the sum of the $E_{s}{/N}_{0}$ values of the three hydrophones, the higher the recognition accuracy.

**Figure 7:**
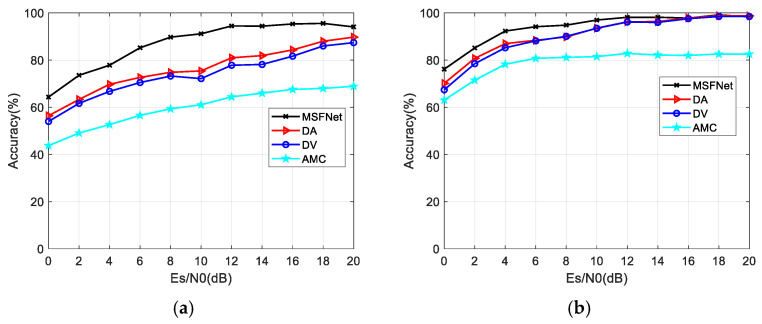
Comparison of recognition performance of different fusion methods. (**a**) M=3; (**b**) M=5.

Next, to highlight the advantages of MHFNet, different fusion methods, including DV, DA, and AMC [14], where the “AMC” refers to the average recognition probability of M hydrophones, are compared. Considering fairness, the same data set is used to train and test all the methods. Figure 7 shows the performance of the different methods. Overall, MHFNet performs better than other methods. Specifically, when M=3, MHFNet has a more flexible fusion mechanism and a higher recognition accuracy than other methods. When the $E_{s}{/N}_{0}$ value is 10 dB, the accuracy of MHFNet is higher than that of DA by approximately 16%. When M=5, the performance of different fusion methods is improved. In the test range of 0–10 dB, MHFNet still has obvious advantages compared with other methods.

**Figure 8:**
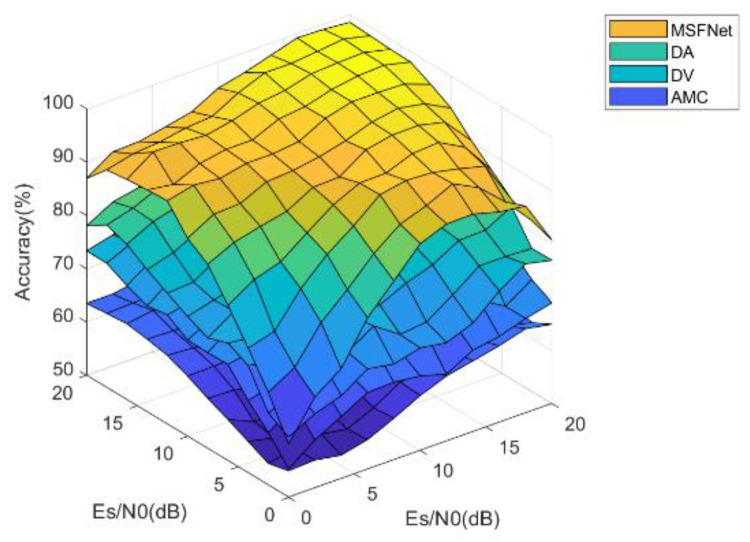
Overall performance comparison of different fusion methods.

In addition, the recognition performance in different receiving scenarios is compared for more comprehensively highlighting the advantages of MHFNet. Figure 8 shows the comparison results of three hydrophones under different receiving scenarios, assuming the $E_{s}{/N}_{0}$ value of a hydrophone to be 10 dB. The three-dimensional graph shows that MHFNet performs better than other methods. The fusion performance advantage of MHFNet is especially obvious when the $E_{s}{/N}_{0}$ values of different hydrophones vary greatly.

**Figure 9:**
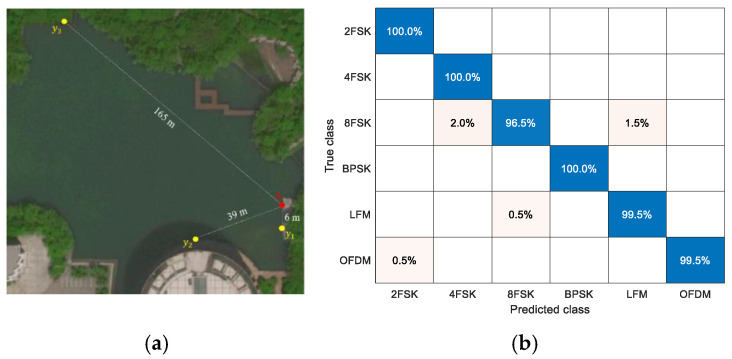
Actual underwater acoustic communication experiment. (**a**) Experimental equipment layout; (**b**) signal recognition confusion matrix and the average recognition accuracy of MHFNet is 99.3%.

**Figure 10:**
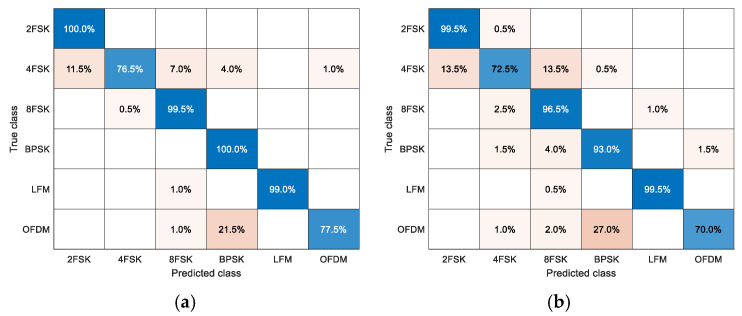
Actual signal recognition confusion matrix. (**a**) The average recognition accuracy of DA is 92.1%; (**b**) The average recognition accuracy of DV is 88.5%.

Finally, to test the performance of MHFNet in the actual underwater environment, we design an underwater acoustic communication experiment in the artificial lake. The weather conditions are as follows: cloudy day, northeast wind level 1, and 17 °C temperature. The layout of the experimental equipment is shown in Figure 9a, where s is the communication sound source, and $y_{1}$, $y_{2}$, and $y_{3}$ represents hydrophones in different positions. In addition, we adopt the two-step fine-tuning method of literature [6], using 30 samples per class per hydrophone to fine-tune the network parameters and 200 samples per class per hydrophone for the test. The recognition confusion matrix is shown in Figure 9b and indicates that each signal class yields a higher recognition accuracy. In addition, we adopt DA and DV as comparison algorithms. To ensure the fairness of the comparative experiments, we use the same fine-tuning strategy and the same data set to fine-tune and test the algorithm. The recognition confusion matrix is shown in Figure 10. It can be seen from the comparison results that MHFNet has better recognition performance. Thus, the recognition performance of MHFNet is further verified.

## 6. Conclusions

This study proposed a modulation recognition method called MHFNet for the underwater SIMO system. MHFNet adopts a feature extraction module and a fusion module to perform feature extraction and fusion on the signals received by multiple hydrophones. The experimental results showed that the fusion mechanism of the proposed neural network is better than other fusion rules. In addition, MHFNet can not only be applied to underwater acoustic communication signal modulation recognition but also provide new ideas for classification problems in other multisensor receiving scenarios.

## Figures and Tables

**Table 1:** Signal parameters.

Signal Types	Symbol Rate (Baud)	Modulation Index	Roll-Off Factor	Carrier/Center Frequency (kHz)	No. of Subcarriers	Frequency Modulation Slope (Hz/ms)
2FSK	{500, 800}	{1, 1.2, 1.3}	/	[15, 16]	/	/
4FSK	{500, 1000}	{1, 1.2, 1.3}	/	[15, 16]	/	/
8FSK	{500, 1000}	{1, 1.2, 1.3}	/	[15, 16]	/	/
BPSK	{1000, 2000}	/	{0.2, 0.25, 0.3}	[15, 16]	/	/
LFM	/	/	/	[15, 16]	/	{60, 100}
OFDM	{31, 56}	/	/	[15, 16]	{64, 128, 256}	/

## Data Availability

Not applicable.
